# Human and digital ecosystems in the modern household

**DOI:** 10.3389/fpsyg.2024.1426804

**Published:** 2025-01-06

**Authors:** Pam Briggs, James Nicholson, Rhian Lukins

**Affiliations:** ^1^PaCT Lab, Northumbria University, Newcastle upon Tyne, United Kingdom; ^2^NorSC Lab, Northumbria University, Newcastle upon Tyne, United Kingdom

**Keywords:** smart homes, Internet of Things, domestic technology, families, trust

## Abstract

Despite a growing number of studies describing the digital ecosystems of the home, few have explored the human component of this ecosystem and fewer have accounted for household and relationship diversity. We asked the inhabitants of nine households to share images of their digital devices and then interviewed them about how the technology was distributed and used, what roles they adopted in relation to the different devices and what boundaries or rules they set up to manage joint use. Following a thematic analysis, we describe (i) the digital components of the ecosystem and their use; (ii) the humans in the ecosystem and their relationships with technology and with each other, and (iii) interconnectedness in terms of joint use and self- or other-imposed restrictions. We use this data to describe dimensions against which households will meaningfully differ and suggest how these dimensions might be used to explore the implications of household and relationship diversity for future smarthome technologies.

## 1 Introduction

The number and sophistication of digital devices found in the home is growing rapidly with the average UK household typically having nine connected devices and recent estimates for the US, more than double this number (House of Commons Library, 2023). Personal devices such as smartphones and smartwatches are still most commonly used, but shared devices (iPad’s, laptops, smart speakers) are commonplace and smart home or ‘Internet of Things’ (IoT) technologies are growing rapidly (including smart thermostats, smart plugs, door cams, etc.). Naturally, adoption of such devices varies widely across the globe, with, for example, over 70% of homes in North America having access to IoT devices compared to less than 10% of homes in South Asia (Kumar et al., 2019). Nonetheless, digital devices of various kinds are now commonplace within the modern household and so it is not surprising that researchers have been trying to better understand the nature of the *domestic digital ecosystem*, defined here as a combination of interconnected users and technologies which collaborate within the home environment.

These explorations have typically focussed upon the technologies, sometimes at the expense of the humans within the ecosystem. It is not uncommon to find a schematic overview of the digital ecosystem in purely in terms of interconnected smart home technologies (Reinisch et al., 2010; Stolojescu-Crisan et al., 2021). However in the last few years, there has been a greater focus on the interplay between humans and technologies, with one significant body of work focussing on the motivations for adoption of different domestic technologies, typically using variants of the technology acceptance model (Almazroi, 2023; Kumar et al., 2023), and another body of work focussing on family dynamics, digital rules and control issues such as the parenting of screen time (Judge et al., 2010; Kirk et al., 2016; Beneteau et al., 2020; Abel et al., 2021; Duckert and Barkhuus, 2021; Furszyfer Del Rio, 2022) There have also been some attempts to integrate these literatures and to get a more comprehensive overview of how both attitudes to technology and family dynamics can shape the digital functioning of the family home (Carvalho et al., 2015). Finally, and unsurprisingly, there have also been a number of recent studies in the wake of the COVID-19 pandemic that address the ways that families have used communication technologies to stay in touch (Judge et al., 2010; Heshmat and Neustaedter, 2021).

Unfortunately, there have been relatively few attempts to integrate these literatures and so literature reviews that focus on smart home technologies (Reinisch et al., 2010; Li et al., 2021; Stolojescu-Crisan et al., 2021) will often be blind to the way that smart phone use plays out within the family (Judge et al., 2010; Terras and Ramsay, 2016). In fact, there has been surprisingly little work that seeks to properly understand the human component of the digital ecosystem, i.e., to understand how the various smart devices present within the home are appropriated to fit the dynamics of a particular household. Further, the limited work in this space tends to focus on the ‘typical family’ and yet households vary enormously, and the make-up of any individual household will have a significant influence on the way that digital work and play is enacted. There are, of course, exceptions, which include a speculative design paper by Oogjes et al. (2018) which specifically pushes the boundaries of what the components of a household might be, work that explores the use of household technologies amongst divorced parents and children in joint custody situations (Odom et al., 2010), a paper that focusses on lower income households (Benton et al., 2023) and a recent study that asked how users from a wide variety of households have shaped their smart home ecosystems, noting that user roles within the ecosystem are dynamic as specific individuals execute ownership and control over particular technologies and noting, too, the critical importance of being able to both share and conceal data from others (Woźniak et al., 2023).

In our own work, we are building on this limited body of work, trying to understand the digital ecosystems across different households to pinpoint the ways in which household differences and dynamics impact smart technology use. We conducted a study with nine diverse households, all of which contained a ‘smart home’ component of some kind to extend our understanding of how household diversity can impact smart technology use. The contribution of this paper is a better understanding of the dimensions against which households to support the development of future smart home technologies.

## 2 Background literature

### 2.1 The human factor in the digital ecosystem

In 2018, the PETRAS Consortium (The UK’s National Centre of Excellence for IoT Cybersecurity) published ‘The Little Book of Design Fiction for the Internet of Things’. In it, three near-future fictions were described: Polly, the world’s first truly smart kettle; Allspark, an energy company providing smart batteries for the household and Orbit, an IoT door lock. For each of these, a detailed description of the relevant technical and service infrastructure was described, with associated threats and opportunities. What was curious about each was the notable absence of people. Users were discussed in the abstract (e.g., in terms of identity verification or guest access) but humans did not populate these digital homes. Indeed, people are quite commonly omitted from descriptions of the smart home ecosystems, which is surprising given that different social values are inevitably negotiated within the household ecosystem and these different values can impact the use and purpose of digital devices in the home (see Kenter et al. (2015) for a discussion of the importance of social values within ecosystems and Ziemba (2019) for a discussion of how sustainability values influence digital adoption).

Often, the ‘human factor’ is limited to an exploration of what influences the *adoption* of new digital technologies. A recent literature review (Kumar et al., 2022) showed that much of this adoption work draws on the technology acceptance model (TAM) (Davis, 1989) and its variants (e.g., UTAUT2 (Venkatesh et al., 2012)). Studies will typically be survey based, using structural equation modelling to demonstrate that a wide range of factors including perceived cost, performance expectancy, effort expectancy, social influence, intention to use, privacy concern, trust in technology, locus of control, age, gender, education, income, etc. can predict the adoption of smart home technologies (Habib et al., 2020; Canziani and MacSween, 2021).

People are often foregrounded when households are discussed in terms of their everyday digital communication practices. In such circumstances, social media or smartphone engagement is often a focus (Terras and Ramsay, 2016) and issues such as screen time become topics for debate and discussion (Kaye et al., 2020; Duckert and Barkhuus, 2021). Some of this work has looked specifically at nuclear families - exploring the way digital device use is negotiated between parents and children, with studies such as that by Beneteau et al. (2020) describing the way that listening devices could be used both productively but also disruptively within the family home. Other studies have looked at a more dispersed family model, sometimes with a focus on long-distance relationships, or on families divided (e.g., because of the COVID-19 pandemic, or divorce) (Odom et al., 2010; Jenkins, 2017). For example, Abel et al. (2021) conducted systematic review of studies published between 1997 and 2019 that explored how long-distance families use social media to keep in touch They described a range of uses for social media, highlighting some of the bonding outcomes of different social media affordances (e.g., synchronous vs. asynchronous communication, video vs. text) as well as showing how different family rituals, (helping with homework, preparing meals, Christmas celebrations) could be enacted on different forms of social media. Some of the papers in this study (and published subsequently) have dealt explicitly with the way the COVID-19 pandemic affected household activities, as people were forced to work from home and rely upon digital means to stay in touch with friends and family (Heshmat and Neustaedter, 2021).

As noted earlier, a relatively small literature has moved beyond the notion of ‘family usage’ to consider diversity in the make-up of the modern household, but this is considered briefly in the section below.

### 2.2 The human and social composition of the digital household

It has been argued that four major factors affect technology adoption and use within the household (Ziemba, 2016): economic factors (available income and price of technology); technological factors (availability, infrastructure, security, ease of use, etc.), social factors (household demographics and composition, social influence, motivation and perceptions of risk, trust, privacy, etc.), and organisational/motivational factors relating to both hedonic outcomes (enjoyment) and utilitarian outcomes (efficiency, ability to work from home). In this study, we are particularly interested in the ways that social and organisational factors influence the resulting ecosystem and the dimensions of diversity that give rise to different patterns of household use.

Turning firstly to social factors, then one simple but critical issue concerns the make-up of the household and the relationship between household members. In a family setting, two or more generations may co-exist, bringing into play issues around age, parental control, digital experience and expertise. Let us take a simple example and explore the impact of age, which is often seen as an important factor when considering the adoption and appropriation of digital devices. Younger people are often seen as early adopters (Cannizzaro et al., 2020) and in many households may have a role in not only pushing for the purchase of certain devices, but in their set up and management. As early as 2000, Sarah Kiesler and colleagues documented the rise of the teen guru within the family—a young person able to offer technical help and support to older family members (Kiesler et al., 2000). They noted, not only the ways that adults turned to teenagers for support, but also the ways in which teens were instrumental in lobbying for computers within the home—and subsequently used them primarily for play. At times the adults were uncomfortable with this power structure but accepted it as the cost of getting effective computing that would satisfy their more instrumental needs. The teen guru is given less prominence in recent years, having perhaps been eclipsed in the literature (somewhat paradoxically) by papers describing the ways in which parents seek to control device use (and screen time) of their children (e.g., Bruun et al. (2020) and Kaye et al. (2020)). However, recent work has made an argument for more democratic householder participation when it comes to who has digital oversight (Adeyeye, 2023; Benton et al., 2023) and this includes the possibilities of pooled oversight in extended households (Akter et al., 2023).

Let us consider what happens when peers share a household (as, for example, in the case of a student flat share). Under these circumstances, one might assume that personal information (pertaining to health or finance for example) would require greater protection. Yet whilst there is a literature that focusses on the ability of individuals to properly secure home devices (Hung et al., 2016) there is relatively little discussion of the human relationships involved especially around issues of interpersonal trust. We do know that device sharing is commonplace within households (Jacobs et al., 2016; Matthews et al., 2016) and that sharing protocols are essential for smart locks and other household security measures (Jha et al., 2019). However, very little is understood about the extent of ‘insider threat’, where a trusted member of the household accesses an individual’s private data without their consent. Within the home environment, there is often an assumption of trust between household members, sometimes to the point where security is simply taken for granted (Watson et al., 2020). Yet it has been found that at least 1 in 5 adults have looked at another individual’s phone without consent (Marques et al., 2016) and again, we need to understand how the trust dynamics play out in a household, as a group of friends are likely to respond very differently to a group of strangers who have been allocated a place together. And even in the most trusting of households, what happens when a visitor joins?

Taking Ziemba’s (2016) categories as a starting point, we can begin to see a number of key dimensions on which the human ecosystem in a digital household will differ: in size, demography, expertise, authority and power, sociability, trust, privacy needs, motivation, usage patterns, etc. Some of these factors were investigated in the recent paper by Woźniak et al. (2023) on smart home ecosystems and to a certain extent we are building on this paper in our study. The authors recognised the need to look at household diversity in their study, interviewing 20 participants about their lived experience within the smart home. The number of devices in each home varied from three to over 30 and the make-up of the various households was also diverse—many participants lived with partners, some with children, some living with their parents and others co-habiting or living alone. The authors captured the motivations and benefits for buying and installing devices and outlined the kinds of roles household participants may adopt (administrators or active/passive users) and the different conflicts that may arise from these different roles. They also recognised the intentional and unintentional interconnectedness of devices, linking this to issues of trust and control and noting that while household members would be relatively happy to share devices with each other, they were more reluctant to exchange and merge their data, something that resonates with an earlier study on device-sharing (Matthews et al., 2016).

The Woźniak et al. (2023) study makes an important contribution to our understanding of the different digital ecosystems that develop, however, they have only begun to scratch the surface in understanding the ways in which households differ from one another. They conclude by focussing on design implications from their work. Rather than focus on design, *per se*, we are interested in the ways in which different households give rise to a variety of interconnected relationships and activities that may, or may not, be supported by technology, something given careful consideration by Sørensen et al. (2014). In our study, then, we aim to capture the way relationships and activities are enacted on a human-technology ecosystem, trying to capture the various dimensions on which households differ, and offering up ways for designers to conceptualise diversity in the digital household.

We interviewed the members of nine smart-home households, choosing to interview them collectively and *in situ* (as opposed to conducting individual interviews with single household members). As well as documenting both the human and technological makeup of the home, we also asked questions about household roles, about how when and where digital devices were shared and questions about the interconnectedness and control of joint devices. Our contribution is twofold. Firstly, we add to the very limited literature that looks at household diversity in the digital home ecosystem and secondly, we offer a structured means of understanding this household diversity in terms elucidating the important dimensions against which households will meaningfully vary.

## 3 Method

### 3.1 Method and materials

A qualitative research strategy was used to explore how households use and govern their digital devices. Nine households were recruited via an advert on social media specifically focusing on homes with multiple occupants who had at least one shared digital internet-connected device within the household. All potential households were made aware that they would need to share pictures of every digital device used by the participants with the research team during the interview sessions. This was done either through a Pinterest^1^ board dedicated to the household devices or through e-mailing the researcher images of the digital devices. An online semi-structured interview was then carried out with each household with all members present, using the photo inventory as prompts at various points in the interview. All participants in the household took part in the interview as a group to encourage more conversation regarding technology usage, and the different opinions of users in the home. This in turn also allowed us to see more direct interactions between the users, such as generational differences (see 4.2.1). The interview aimed at an understanding of the household setting, how technology is used within the household, technology problems and their solution, the roles of members of the household in relation to the technologies, boundaries and rules of technology use and any trust, security or privacy concerns. All interviews were audio recorded and transcribed for analysis.

### 3.2 Participants

We were interested in recruiting non-stereotypical households in order to explore the different perspectives towards living together, sharing technologies and how this impacts the ecosystem as a whole. The study was advertised through social media posts, aimed at recruiting a variety of different households including house shares and families with at least one parent and one child aged 13 or above. From this, nine households came forward to take part, with a total of 23 participants. Each household had between two to three participants, either being a family household (Households 1; 2; 5; 7), a couple (Households 4; 9), or a houseshare (Households 3; 6; 8). There were 14 male and 9 female participants and ages ranged from 15 and 63. Additional demographic information can be found in Table 1.

**Table 1:** Interview participant demographics, household occupants and IoT devices.

Type of household	Gender (Age)	Occupation	Devices
Household 1 (family)	M (23)	Not in work	Home computer, iPad, 3 mobile phones, laptop, smart TV, HIVE system
	M (50)	Wealth manager	
	F (47)	NHS worker	
Household 2 (family)	M (23)	Store worker	4 security cameras, HIVE System, iPad, 3 smart phones, laptop, smart TV, Alexa
	M (23)	Store worker	
	M (63)	Retired	
Household 3 (houseshare)	F (23)	Student	4 laptops, 3 smart phones, 1 Google home, chrome cast, Alexa, Wii, Apple TV unit, Google lamp, smart TV, home desktop
	F (23)	Florist	
	M (23)	Health tech administrator	
Household 4 (couple)	M (23)	Army	2 smart phones, smart TV, iPad
	F (18)	Army	
Household 5 (family)	M (20)	Apprentice Bricklayer	3 smart phones, PS5, Xbox 360, Home desktop, iPad, Sky TV unit, laptop, 2 smart TVs
	M (58)	Support worker	
	F (53)	Secretary	
Household 6 (houseshare)	M (22)	Masters student	2 smart phones, 3 laptops, smart TV, PS4, Wii, smart watch, Google home
	F (22)	Support worker	
	F (23)	Support worker	
Household 7 (family)	M (49)	Lecturer	2 Macs, iPad, 2 iPhones, Google nest, Alexa, Kindle, Nintendo switch, smart watch, smart TV, PS4, laptop
	F (15)	Student	
Household 8	M (23)	Support worker	PS5, PSVR, PS4, 2 smart TVs, Apple watch, tp link Wi-Fi system, 2 Google homes
(houseshare)	M (25)	Part-time researcher	
Household 9 (couple)	M (24)	Office worker	2 smart phones, iPad, PS4
	F (24)	Teacher	

### 3.3 Interview procedure

Before the interviews took place, participants provided the researcher with a photographic inventory of the digital items in the home, sent via email or via a shared Pinterest board. Participants were asked to include anything that was digital that was either used by one person within the household or was shared by multiple users. Images ranged from smart phones to smart TVs and smart home hubs/assistants. This inventory was referred to multiple times within the interview and allowed the participants to draw upon real experiences shaped by their digital devices.

The first part of the interview involved discussion about the general workings of the household. Prompts such as “What do you all do (i.e., work, school etc.)” helped inform the researcher about the nature and relationship of people being interviewed. In part two, the interview explored the use of devices in more detail and included questions such as, “do you find there to be any downsides to sharing technology?” and “is there much shared data in your household.” Part 3 of the interview concerned how participants faced the problems and difficulties proposed by their digital items. Questions such as, “How did they affect you?” and “How did you deal with them?” began discussion centred around how resilient participants were to cyber related problems. Additionally, questions such as “Do you usually solve problems yourselves when you discover them?” and “Is there one person in the family who usually helps people?” helped the researcher understand the hierarchy of responsibility within the household and which members were the ‘go to’ people for problems.

Part 4 of the interview schedule centred around the topic of the boundaries and rules set within the household. Within this section, the researchers could expand upon the hierarchy of responsibility within the household and probe participants into the specific rules set by dominant users. Questions such as, “How much do you set rules on what should and should not be done online or with technology?” explored how participants restricted themselves and others to prevent possible issues with security and privacy problems. The final part of the interview asked how house members dealt with security and privacy issues. The interviews ranged from 26 min to 1 h 28 min in length. After the interview was conducted, participants were debriefed, and each member given a £10 Amazon voucher for participation.

### 3.4 Analysis

The interviews were transcribed and formed the basis of a codebook approach to thematic analysis (Clarke and Braun, 2017) in which the researchers firstly familiarised themselves with the data through initial read-throughs of the transcripts and collaborative discussions surrounding potentially interesting data points and narratives, informed by existing frameworks from Ziemba (2016) and Woźniak et al. (2023). Following three sessions of discussions, the lead author began a primarily semantic coding process where relevant quotes were labelled and recorded within a preliminary table and clustered into meaningful themes. The themes were checked through collaborative discussions with all three members of the research team, allowing different perspectives to be taken into account. Once an interpretation of the themes had been agreed, appropriate labels were applied.

## 4 Results and discussion

Following the analysis we report on eight themes, grouped into three topic clusters: *technologies in the ecosystem*, including the drivers to buy and use those technologies, *humans in the ecosystem*, their demographics, relationships and roles and *interconnectedness* in terms of the way shared devices are managed and boundaries are set, see Table 2. Note, in the text below quotes are followed by an indication of which participant spoke and from which household (refer to Table 1 for details).

**Table 2:** Topic clusters and themes.

Topic cluster	Themes
Technologies in the ecosystem	Negotiating adoption and use of different devices
	Financial considerations
	Forms of technology dependence
Humans in the ecosystem	Age and generational considerations
	Administrative responsibilities
	Trust relationships between household members
Interconnectedness	Togetherness
	Rule setting

### 4.1 Technologies in the ecosystem

This section discusses the range of technologies in each of the households and how easily users were able to adopt these into their everyday lives. The number and usage of digital devices varied considerably depending on the requirements of both the household and the individual users. Many individuals talked about working from home, although desktop computers were only found in two households. House-sharing adults had more shared devices, often games consoles, which were used to bring everyone together on an evening (see section 4.3.1). All households had at least one device for entertainment, mainly smart TVs and games consoles, while six used home automation technologies, such as Amazon Alexas and HIVE heating systems. One household had security-specific smart technologies in place. The most technologically saturated household reported 16 devices, the largest number in the study and higher than the UK average of two smart devices per person (Cannizzaro et al., 2020), but this might be typical of houseshares, where different adults have bought their own devices.

#### 4.1.1 Negotiating adoption and use of different devices

Utilitarian factors such as convenience, perceived usefulness and ease of use are often critical motivators for the adoption of various digital technologies (Habib et al., 2020; Kumar et al., 2023), whilst hedonic (enjoyment) factors also play a role. A critical issue here, where there is relatively little research, is the way in which device purchase and adoption is negotiated between members of a household or is left as an individual decision.

For our households, adoption decisions had been clearly affected by the COVID-19 pandemic and subsequent lockdown practices. We know that users across the globe quickly adopted video (e.g., Zoom) and security (e.g., VPN) technologies when working from home (Pranggono and Arabo, 2021) and unsurprisingly, our participants were able to reflect on the additional reliance on technologies in our interviews.

*“It just shows you how you need it more, especially at this moment”* (P3, H1, Family).

*“Since COVID I transitioned to a different role but also working from home and that was sat on a desk in front of a computer for 8/9 h a day”* (P2, H1, Family).

The impact of the COVID-19 pandemic on digital engagement has been widely discussed, not only in terms of changes to hybrid working practices but also in terms of increased reliance upon digital communication, even within families (Vlachantoni et al., 2023; Zapletal et al., 2023) and so a reliance upon digital technologies is to be expected.

More controversial was the adoption of digital assistants such as Alexa. Here, the tension between convenience and intrusion played out in household decision making where, for example, within H3 (a houseshare), one individual (P3) disliked the ‘creepy’ nature of Alexa but the household continued to use it, because another member (P1) enjoyed the hedonic benefits of Alexa (primarily using it as a voice-activated speaker to play music), but also enjoying the way that control could be shared amongst friends:

*“I think with the Alexa it is nice because we have a party night and it is nice to have that shared, everyone is listening to music together and that is really nice - so we all pass around the phone and we will all queue songs we each want to listen to. Obviously, the entertainment you get from watching the tele all together is a nice upside”* (P1, H3, Houseshare).

Some users adopted digital devices as a kind of ‘comfort blanket’ which meant that in some households, certain individuals wanted TV, music or radio to be ‘always on’, whereas others would prefer more selective use.

*“I mean I probably rely on <technology> a bit more than <household member>, mine is more of a comfort thing. It is not so much I’m engaging with it like a TV show, I just have it on so it’s not silent”* (P2, H9, couple).

*“If the TV is not on then I cannot get to sleep”* (P1, H2, Family).

Both users, aged 24 and 23 respectively, use their smart devices to provide a background noise to create a more comfortable, relaxing atmosphere and indeed, recent literature has shown that the use of technology as a comfort blanket is common amongst younger users, and related to stress reduction (Pressman and Hunter, 2011).

#### 4.1.2 Financial considerations

As new technologies continue to become available on the market, users may choose to upgrade their old devices to a newer model, or to buy the next best thing to upgrade their home automation or entertainment set up. When looking to purchase these devices, users will be met with an initial cost of the device itself, which generally start at around £40 (Felstead and Reuschke, 2020). However, many users may not be aware of the other charges associated with smart devices, such as the initial purchase, installation, subscriptions, maintenance and service charges (Kumar et al., 2023). For example, while a wired Ring doorbell will cost £90 upfront, installation will be £160 and the advanced features will cost an additional £24.99 per year (CheckATrade, 2023). It makes sense, then, for those who share a home to also consider cost-sharing, although those cohabiting on a temporary basis obviously benefit more from subscription sharing, as issues of ‘owenership’ are avoided.

*“When you share technology, especially when it’s something with a subscription, then when you are sharing with 3 you can third the price.”* (P2, H3, Houseshare).

*“I’m set up on someone else’s account, I did not have Netflix. They wanted to charge me £9 a month and my daughter said I’ll set you up. So, on my TV I can put my Smart TV on and I can watch all these movies for free”* (P2, H2, Family).

Many of the household participants discussed sharing their technology with others in the home, allowing all members of the household to watch the TV or use the games consoles without issue. This also allowed the users to share the cost evenly between those in the home, which would cover the initial fees associated with the device as well as the maintenance and service costs later down the line. In some cases, it meant introducing housemates to new devices they had previously ignored:

*“I never had a PS4 or anything moving into this house and playing on the PS4 and learning to enjoy games so like yeah – it’s a new form of socialising which is nice”* (P3, H6, Houseshare). Device sharing is a widely recognised benefit of household membership, and these forms of sharing are not uncommon, particularly when they help to save money. Matthews et al. (2016) have noted that such sharing practices are not only driven by financial considerations, but can critically rely on trusted relationships between the sharers (something we consider in more detail below). That said, even in a home where trust is high, individual members may have distinct needs and negotiations are inevitable as to who in any household might take precedence (see Lee and Lim (2024) for a discussion of such coordination issues within families). Given that the individual that pays the bill is likely to also have some kind of executive control of a device, then the impact of differential income levels can be important, particularly for members of a houseshare.

#### 4.1.3 Forms of technology dependence

Participants recognised how much of their lives was now dependent upon technology and realised the devastating effects of power cuts or other interruptions.

*“We’ve got, for instance, our heating is all through the HIVE app. Which is on my phone, I’m the online one with it on my device. So, I control it when we are out” (P3) “If we had no internet, we’d have no heating”* (P2, H1, Family).

*“When there is a power cut or something you realise how important they are. You do not have Wi-Fi or chargers”* (P2, H7, Family).

Regardless of the device, many users spend most of each day using technology for work, education, communication and for entertainment without realising the length of time spent doing so. Some participants expressed the view that a life with technology is all they know, e.g.*, “We rely on it, we do not know anything else”* (P2, H2, Family), whilst others recognised that levels of technology use could be considered problematic:

*“I would not use the word addicted, but reliant – I cannot imagine without it, I certainly could not do my job and you would feel incredibly isolated. It would impact your wellbeing”* (P1, H7, Family).

Reliance upon digital devices seems to be a facet of modern life, and, of course, there has been a huge debate in the literature around the costs and benefits of ‘screen time’ within the home (K. Kaye et al., 2020). Again, this literature has played out in the family context, where much of the work has focussed upon the screen time of young children. However, it is notable that there has been an increased attention to the way that adults might seek to limit excessive use of smartphones, sometimes with the intervention of digital wellbeing apps (Roffarello and De Russis, 2023). It is interesting to note similar debates emerging from diverse households in our sample, and we return to this point in our discussion on self-policing, below.

### 4.2 Humans in the ecosystem

By interviewing the household occupants as a group, we were able to pinpoint the ‘hierarchy’ in terms of technology adoption and installation. Alongside this, we discuss how trust is affected when sharing devices, and how age may affect usage and understanding.

#### 4.2.1 Age and generational considerations

The demographics of households differed and where households were home to different generations of users, we would see age cited as a key factor affecting technology use. Many expressed views in keeping with received wisdom about the relative advantages of the younger ‘digital natives’ who had been raised with the technology:

*“Daughter has always grown up with technology, she has always had it around since she was little, so it rolled into the fabric of our lives”* (P1, H7, Family).

*“[Mam] got a smart hoover and she was getting upset cause it was broken but I went in, and I just pressed a button. Even if our generation do not understand something we can find it out”* (P2, H7, Family).

*“Because they are the younger generation, they do everything online. Compared to when we were younger, it was like a brick computer”* (P2, H1, Family).

Researchers have established that age is a factor in predicting the number of devices in use (Cannizzaro et al., 2020) with those aged 65 and over generally adopting fewest devices. Our households did not include anyone over the age of 65, something to take into consideration in future work, but we can see here that stereotypes of older adults mean that people will often assume that adults are less open or adverse to new technologies and are ultimately unwilling to try new technologies (Olson et al., 2011; Morrison et al., 2023).

*“They’re just not up to date, elderly people or people up to date with phones”* (P2, H4, couple).

It is important to note that these stereotypes overlook the fact that many older individuals are highly competent both with smartphone use and with cybersecurity practices (Morrison et al., 2023), and that many older adults became skilled in the use of digital technologies following the COVID-19 pandemic (Vlachantoni et al., 2023). Yet it is interesting to see how easily stereotypes are adopted by both younger and older individuals throughout our interviews. As Birkland (2024) has noted, stereotypes about older adults’ technology capabilities are pervasive and can be easily assimilated even by those older people with high digital competencies, and so it is interesting to see some of the ways in which these assumptions play out in the home context.

#### 4.2.2 Administrative responsibilities

Another key difference between households was the distribution of expertise, which was particularly important when technical problems occurred. In some cases, household members would simply use Google to try to solve a local problem:

*“It kind of depends on how much I like the technology and how easy fixable it is. First port of call is off and on again, second is google it, third is can I live with it, if I can live with it then I’ll suck it up. If I cannot then it’s well worth repairing”* (P1 H9, couple).

In most cases, one individual within the household becomes known as the expert—described by Woźniak et al. (2023) as the ‘household administrator but also referenced by Rode (2010) as Security Czars. In some cases, this is a parent or knowledgeable adult, someone who introduced the new device into the home and has taken on responsibility for installation and maintenance (Woźniak et al., 2023). In other cases a child or junior member of the household will simply take control:

*“Tech support, that’s my youngest’s middle name”* (P2, H1, Family).

*“… all my kids. Luke sets my TV up, my phone up, my cameras up … all him”* (P1 H2, Family).

Note that expertise is not always the key issue here, as some participants recognised the need to simply be calm and seek appropriate external advice:

*“I think you would rely on me, you aren’t level headed enough to deal with it… I am just more level headed and I can think things through rather than going from 0–100 you know, get in touch with the right companies, explain the situation and try and get a solution”* (P1, H4, couple).

Such dynamics are interesting, as it makes it difficult to make assumptions about who will be taking on the role of household administrator—whether it be the user who has the most interest in technology, the most experience, or simply the person who can remain the most calm under pressure. It is also interesting that throughout our interviews younger people were delegated digital responsibilities, whereas in earlier work this role was assumed predominantly by male adults who kept close control over younger members of the family (Rode, 2010). Work on *digital housekeeping* has proliferated recently, triggered by an understanding that our household devices represent a burden that must be managed. However, in most discussions, such digital housekeeping is seen as a matter of taking personal responsibility for digital decluttering (e.g., Horst and Sinanan, 2021). It is interesting to note that our participants did not refer to the mess of household data, perhaps because data is not considered a shared commodity, but restricted their housekeeping discussions to the devices themselves.

#### 4.2.3 Trust relationships between household members

We have seen that the technology ecosystems contained a mix of shared and personal devices and one important human factor was the extent to which the personal devices or logins were shared by other members of the household. This boiled down to an issue of interpersonal trust:

*“I do not restrict access to my data to anyone in the household, like if they want to look at my photos, I’ll hand them my phone. I do not really have a separation to data and things. There are some things with my work that I would not be happy if they accessed it, but with my household members I could give my laptop to them for 24 h and I would not be concerned about what they were doing on it.”* (P1, H3, Houseshare).

“I have my credit card on my PS5 but I do not think anyone would go on there and do anything like buy things because I trust the people I live with. (P2, H8, Houseshare).

*“[trust] was never an issue, unconscious boundaries so we do not look in each others phones for anything because we have never needed to and that has not changed. We are a bit more interchangeable I guess, not to a massive extend by with our devices. You are happy to use what is your PS4 because well what am I gonna do. There is nothing on it that is, or like that is mine you cannot use it”* (P2, H9, couple).

In all of these cases, there is an assumption that a similar, high level of trust operates throughout the household (a houseshare between friends and one is a young couple) and yet it may clearly be the case that trust can exist between some individuals, but not between others. In a 2016 study, researchers found that 31% of their participants had looked through someone else’s phone without their permission, often for the purpose of finding out sensitive information (Marques et al., 2016) which means that users are opening themselves up to data breaches and potential identity theft. Clearly the young adults in our sample felt comfortable with such data sharing although we should note that in our sample we have limited data on trust within family households, where a known issue in the literature is the parental mistrust of teenage children’s use of digital technologies (Wisniewski et al., 2014).

We should note, here, that interpersonal trust plays out in interesting ways when we consider household administration. As discussed earlier, the ‘administrator’ is often one trusted individual, yet we know that, when trust is low, or when other power or expertise differentials are high, tensions can arise (Geeng and Roesner, 2019). Apthorpe et al. (2022) discuss what can happen when individuals have access to privileged data and when there is low transparency between household members, leading to suspicion and a gradual erosion of trust between members of a household, noting that this is a particular problem when co-habitants know each other less well.

### 4.3 Interconnectedness

Throughout the interviews the participants began to discuss the serious side of sharing technology in a household, particularly in terms of when and how they might be used collectively and how the rules of use and access are negotiated.

#### 4.3.1 Togetherness

Although every household member in our sample owned a personal digital device, there were many shared devices across the households, such as smart TVs, smart speakers and games consoles. We found that households were using technology as a focus for social communication, rather than to isolate themselves from others: While spending time together can vary depending on routines, who in the home is available, and the specific needs of the household, time spent together was often digitally mediated:

*“It is mainly social. The technology brings us together even if the TV is on we are talking, Alexa is good cause we pick songs. It adds to the ambience”* (P3, H2, Family).

*“It generally tends to happen on a Friday night when everyone’s winding down … We’ll just put the music channels on, and it tends to draw us together”* (P3, H1, Family).

This use of technology as a focus for social communication is interesting, as it contrasts with existing literature reporting on digital devices being the cause for member isolation in both parents and adolescents (McDaniel, 2019), and instead sheds light on how households use devices collaboratively.

TVs and smart speakers are among the most mentioned devices used to encourage spending time together, as it can provide entertainment in the form of a new TV series, or background music while dinner is eaten. This point is reinforced by P1 HI who states:

*“I mean I’d say one of the most important ones is the TV because it brings us all together. Whereas everything else kind of individualises us”* (P1, H1, Family).

An interesting point about ‘together time’ was the need to curate what was watched or what games were played and our participants would often describe their willingness to compromise if it meant that they could spend quality time together. This was true of both families and houseshares:

*“I guess the nice thing about only having one PS4 to watch telly means that we do watch things together even if you are watching the football and I do not care, I will sit in here and watch it too so we are spending time together”* (P2, H9, couple).

*“So, when we do watch TV together which is the most common, joint activity, we tend to curate it if you like, watch specific shows rather than watching whatever is on”* (P1, H7, Family).

*“I might want something on the TV a bit different to x and it’s like oh we’ll watch this for you and then this for me … so like you chose last time so I chose this time”* (P3, H6, Houseshare).

Given the screen time literature described above, examples of positive use of screens is interesting and plays into the debate around the pros and cons of technology use (Kaye et al., 2020). We should also be mindful that spending time on individual devices may imply a different form of togetherness with distant others. Our participants recognised the importance of being able to use technology to maintain more distant family and friend relationships, particularly following the COVID-19 pandemic (Heshmat and Neustaedter, 2021):

*“I am not so dependent on social media because I’m not interested but to keep in contact with family back home. I cannot just go back to home to see my family because it’s like 300 miles away”* (P1, H4, couple).

This need for togetherness with distant others can affect family members, but we should be mindful that it is likely to be a stronger issue for student or temporary housemates who will often rely on virtual means to maintain home ties (Hiller and Franz, 2004). An important factor when considering diverse households, then, is the ease with which individuals can reach out to those closest to them, even over long distances. Togetherness as a more global phenomenon is considered by Wang et al. (2024) who note the strange role of the smart speaker in bringing family members together ‘back to the living room’ for shared activities, similar to the music sharing described above by our participants, whilst at the same time offering the instant capability of global connectivity.

#### 4.3.2 Rule setting

In order to maintain some form of communication and time spent together, households create rules for device use, some of which were enforced by authority figures (typically within a family setting). One of the most common examples of these imposed rules was not allowing smart devices to be used when users ate together.

*“If we come round and we eat I make everyone turn their phone off. Kids sit and talk and eat and they are all on their phone and I think that’s rude. If we are in a restaurant the first one to pick their phone up pays the bill”* (P1, H2, Family).

*“I would say no devices when we are eating our tea so we can sit around the table and talk”* (P3, H5, family).

By regulating their technology usage during meal times, members of the household are able to spend more time together as a family unit without it being interrupted by a text tone or videos playing in the background. These rules are common across family households, with many parents choosing not to eliminate the use of these devices entirely, but rather to limit them to set times or for specific functions (Beneteau et al., 2020).

All households discussed the ‘rules’ that they had in place in the home, though many of these, including the examples below, were less strict than some of those imposed in family households.

*“Also making sure things are turned off at the end of the night like with the PS5 controllers”* (P2, H8, Houseshare).

*“We are probably less strict on the rules now, we basically said no tele before nine o’clock but that was for the bills”* (P1, H9, couple).

Within family households, users are still *“expected to be responsible with <technology>”* (P2, H7, Family), though they often have stricter rules regarding access. In the quote below, P1 explains the restrictions they place on their router so that their 15-year-old ca not access inappropriate material, such as self-harm and gambling which may be detrimental to their mental health.

*“I prefer <P2> to be of her phone half an hour before she goes to bed. I think on the BT account I think I have banned some classes of websites. Drugs, self harm and gambling I have filtered those. Not drugs as in drugs awareness but stuff like, more obvious things. Gambling, I do not think she has a problem with gambling and I do not either but I think I just have switched it on by default. We do not have any hard and fast rules about right you have a maximum of an hour a day”* (P1, H7, Family).

Other households recognised that self-policing was the preferred strategy:

*“But <son> we can come up and he’s playing on his games and then the next time we come up he’s got his guitar out. So we know that he knows the balance. So we leave him to his own devices. And something that I’ve started to try and implement now, is from 9 o’clock I try not to look at my phone. I try to keep it down”* (P3, H1, Family).

Although family or other members of the household could suggest that users take a break from technology for a while, this decision is often made by the individual without the need of a push from others in the home. A 2020 study found that many households set silent rules in place, which although were not discussed, were accepted by those in the home. These rules suggest that all users are responsible for their own device usage (Watson et al., 2020) and so it is interesting to note that our participants tended to focus on policing as a communal, rather than a personal act. The absence of observations around self-policing is interesting given the preponderance of new apps that are designed to support digital wellbeing by stepping away from the smartphone (Roffarello and De Russis, 2023), but we heard no discussion on the use of such systems.

## 5 Dimensions in which households vary

In this study, we set out with two main aims: Firstly, to understand more about the interpersonal elements operating in a domestic digital ecosystem and secondly, to do this with a diverse selection of households that would give a better understanding of the dimensions against which households will vary. We consider this important, not only as a step in bringing diverse literatures together, but also as a means of thinking about the development of future smart home technologies.

In reviewing different papers that addressed smart home adoption, for example, we noted the preponderance of economic, technology, social and motivational factors (Ziemba, 2016) and these are reflected in our own data, however, the interplay between these factors and the composition of a household is often missing from the debate. To take a simple example, in a houseshare, people anticipate living together shorter-term and will also bring their own devices. This implies less willingness to invest in longer term technologies (e.g., around energy saving) which means some systems may be absent, but also means that some systems (e.g., smart speakers) may be duplicated. Further, the financial considerations in a student home will be markedly different from those in an established family residence, the levels of trust between household members will vary and the extent to which control of devices is ceded to one ‘administrator’ will also differ. In the section below we try to capture some of these household differences in a more systematic way, detailing some of the dimensions against households may differ.

We note, however, the limitations of our study, where we have only scratched the surface in terms of both diversity and complexity of digital households and we recognise that our small sample was not ethnically diverse, nor did we include non-nuclear families or those living in isolation. Indeed, there are many ways in which households may meaningfully vary, but rarely is this fully discussed.

### 5.1 Variability in digital households

When considering the ways in which digital ecosystems differ, we might begin with four dimensions related to Acquisition and Long-Term Planning (planned vs. *ad-hoc*), Patterns of Use (shared vs. isolated use of devices), Trust between household members, and the Distribution of Digital Expertise.

#### 5.1.1 Acquisition and long-term planning

The smart-home ecosystem literature will often assume that adoption decisions are planned (Li et al., 2021), yet we can see from our sample that the acquisition of devices can sometimes be chaotic, for example, the introduction of an Alexa in H3 (see 4.1.2). The addition of devices was rather piecemeal in some houses (typically house-shares) and more carefully planned in others (typically family homes). This is to be expected: a family home can afford the luxury of longer-term planning (although this can be complicated when adult children are involved) whereas friends (or in some cases strangers) coming together to share accommodation may bring with them a proliferation of (sometimes duplicate) devices. They may adopt a more planned approach towards device adoption over time, but then, as individuals leave the home, problems may arise around device ownership and disruption to formerly ‘shared’ services.

Naturally household finances impact not only on the purchase of digital devices and digital subscriptions/services but also on their maintenance. In family households, an expectation is that parents would have the financial power to dictate the purchase and management of digital devices and services, but family dynamics can be complex and in multi-generational households, seniority and wealth may not necessarily go hand in hand. Financial management of devices in house-shares were conducted on a more *ad-hoc* basis but housemates developed a number of strategies to economise, e.g., with streaming services.

#### 5.1.2 Patterns of use

In any household there will be a balance between shared and personal use of devices that may, or may not, relate to the ownership of those devices. Not all members of a household will agree on how ‘shared’ technology may be used and inevitably conflicts will arise if some individuals need ‘background noise’ and others prefer silence. In all our homes, occupants enjoyed shared digital events involving TV or music and these were sometimes co-ordinated around scheduling of programmes or regular nights together but also happened on an *ad-hoc* basis. Naturally, households will differ in terms of the amount of time inhabitants will wish to spend together and this will change over time. In a family home, time spent alone might vary as a result of the age of the child or the working patterns of the adult, whereas in a house-share, students thrown together for the first time may seek periods of isolation, whereas friends moving in together may be more inclined to occupy shared-space and be more playful with shared devices. Participants in our sample were not vocal about use of devices in isolation, but clearly people were keen to find private spaces to be able to engage in home-working and parents were careful to monitor the amount of time children spent using personal devices whilst isolated (e.g., in their bedrooms).

#### 5.1.3 Trust

Trust dynamics were hard to predict from the household demographics alone. In all households, some devices were communal (e.g., TVs), however, all would involve personal ‘ownership’ of streaming services such as Netflix, which meant that individuals would share login details with others. Family homes could include a variety of exclusively shared devices for family use and some personal devices that could be shared for specific purposes (e.g., web browsing, viewing videos, etc.), but parents would not necessarily have complete trust in their children (Terras and Ramsay, 2016). Houseshares might be populated by individuals with relatively low trust which would mean that personal devices would only be shared (and monitored) for explicit purposes, yet in our study, friends living together were very relaxed about handing over smart phones and sharing passwords for devices and services alike (see section 4.2.3). This is interesting given that privacy and security concerns are often paramount when considering trust in smart-home technologies (Cannizzaro et al., 2020) yet such models do not always take into consideration inter-personal trust as a complicating factor.

#### 5.1.4 Distribution of digital expertise

As discussed previously, many households navigate problems with digital technology through a ‘household administrator’ who takes ownership of running and fixing home digital devices (Kiesler et al., 2000). However, we must acknowledge that not every household will have an administrator, nor will all administrators be adept at handling technical problems. While some households rely on tech-savvy or younger members to be the administrators (Kiesler et al., 2000), in contrast to other work highlighting Security Czars (Rode, 2010)—available in family homes and possibly student homes—others may have less knowledgeable individuals or simply be less confident individuals. Houseshares may have to rely on temperament or relationship status as the indicators available for seeking technical support. Older adults in multi-generational households may seek technical support from younger members, but may not necessarily be willing to cede control (i.e., formally hand over administrator rights). Where there is a digital divide in households, then different dependencies will develop which can be managed effectively or can prove disruptive. In households with low expertise, technical problems could pose serious concerns and result in turning to inadequate sources for help and advice (Nicholson et al., 2019).

## 6 Conclusion

In the user-centred design literature, it has become established practice to design for a variety of users, and yet there is limited evidence of this philosophy in the IoT or smart-home literature, where we see relatively little recognition of household variability. In this study, we have tried to take into account the reality of digital life in a variety of households, noting both the digital and human elements of the different ecosystems and the ways in which people and technologies interconnect. We also noted some of the key dimensions against which households differ, particularly in terms of the ways that devices are introduced into the home, their patterns of use, the levels of interpersonal trust between household members and the extent to which some individuals naturally emerge as experts or ‘household administrators’. These issues are rarely discussed and yet carry important implications for issues such as privacy and identity management, the sharing of administrator rights, device security and indeed the quality of life for household members. However, we should close by recognising that these are not the only ways in which households may differ. Critical issues around household transience or household income are becoming recognised (Benton et al., 2023) and an interesting new literature that focusses on the democratisation of the digital household is also emerging (e.g., (Akter et al., 2023)). These developments are set to add immensely to our understanding of the evolution of home-based digital ecosystems.

## Data Availability

The datasets presented in this article are not readily available because qualitative data can potentially be identifying, despite our efforts to anonymise it for analysis. Requests to access the datasets should be directed to james.nicholson@northumbria.ac.uk.

## Footnotes

^1^ https://www.pinterest.co.uk/
